# Assessing the Role of Muscle Protein Breakdown in Response to Nutrition and Exercise in Humans

**DOI:** 10.1007/s40279-017-0845-5

**Published:** 2018-01-24

**Authors:** Kevin D. Tipton, D. Lee Hamilton, Iain J. Gallagher

**Affiliations:** Physiology, Exercise and Nutrition Research Group, Faculty of Health Sciences and Sport, University of Stirling, Stirling, Scotland

## Abstract

Muscle protein breakdown (MPB) is an important metabolic component of muscle remodeling, adaptation to training, and increasing muscle mass. Degradation of muscle proteins occurs via the integration of three main systems—autophagy and the calpain and ubiquitin-proteasome systems. These systems do not operate independently, and the regulation is complex. Complete degradation of a protein requires some combination of the systems. Determination of MPB in humans is technically challenging, leading to a relative dearth of information. Available information on the dynamic response of MPB primarily comes from stable isotopic methods with expression and activity measures providing complementary information. It seems clear that resistance exercise increases MPB, but not as much as the increase in muscle protein synthesis. Both hyperaminoacidemia and hyperinsulinemia inhibit the post-exercise response of MPB. Available data do not allow a comprehensive examination of the mechanisms behind these responses. Practical nutrition recommendations for interventions to suppress MPB following exercise are often made. However, it is likely that some degree of increased MPB following exercise is an important component for optimal remodeling. At this time, it is not possible to determine the impact of nutrition on any individual muscle protein. Thus, until we can develop and employ better methods to elucidate the role of MPB following exercise and the response to nutrition, recommendations to optimize post exercise nutrition should focus on the response of muscle protein synthesis. The aim of this review is to provide a comprehensive examination of the state of knowledge, including methodological considerations, of the response of MPB to exercise and nutrition in humans.

## Introduction

Skeletal muscle is a crucially important tissue for human health and well-being [1]. The importance of muscle for locomotion and strength is obvious. However, skeletal muscle also is the largest metabolically active tissue in the body. It is also the largest site for glucose disposal and acts as a fuel reserve for other organs during pathophysiological situations, including fasting. Thus, skeletal muscle is critical, not only for athletic performance, but for healthy daily living and aging. Understanding the regulation of gain or loss of muscle mass is therefore an important consideration for exercise and nutrition scientists.

Muscle proteins are constantly turning over, i.e., broken down (or degraded) and synthesized. The balance between the rates of synthesis and degradation of muscle protein pools, i.e., net muscle protein balance (NBAL), determines the amount of that protein in muscle. In particular, changes in the amount of muscle myofibrillar proteins lead to changes in muscle mass. Moreover, in addition to or instead of, modulating muscle mass, changes in muscle protein synthesis (MPS) and muscle protein breakdown (MPB) also may be crucial for repair and remodeling of muscle proteins following exercise [2]. So, the regulation of these processes is critical for optimal adaptation of muscle in terms of size. Thus, exercise and nutrition interventions that influence rates of MPS and MPB, and ultimately NBAL, have received increasing attention in the last two to three decades [2–5].

The influence of exercise and nutrition on the regulation of MPS is understood far better than MPB [2–5]. There are a number of reasons for this discrepancy. The study of MPB, particularly in humans, is technically much more difficult than MPS [6]. Furthermore, changes in MPS in response to exercise and nutrition have a much greater influence on NBAL than do changes in MPB [7, 8]. Also, the resolution of measuring MPS on a protein level is more readily accomplished than for MPB. Thus, the bulk of the studies attempting to contribute to our understanding of changes in muscle mass and in response to nutrition and exercise have focused on examining MPS [6]. Nevertheless, it is important to delineate the role of MPB for remodeling and repair of skeletal muscle in response to exercise and how nutrition influences these processes. This information contributes to our overall understanding of the metabolic processes behind muscle gains, losses, and repair and remodeling of muscle tissue leading to muscle adaptation. In this review, we will examine our current understanding of the process of MPB and how it responds to nutrition and exercise interventions and its role in changing skeletal muscle mass and adaptation. Thus, we will focus our discussion on data from studies in humans following exercise.

## Systems of Muscle Protein Breakdown

There are three main systems that contribute to the catabolic component of muscle turnover; the ubiquitin-proteasomal pathway (UPP), autophagy, and the calpain Ca^2+^-dependent cysteine proteases. The best known of these processes is the UPP, which centers around the 26 kDa proteasome that degrades proteins tagged with the 8.5 kDa protein ubiquitin [9]. The UPP is central to protein degradation across all cell types and plays a fundamental role in normal physiology. E1 enzymes first activate ubiquitin. These enzymes capture ubiquitin and an ATP-Mg^2+^ complex, and catalyse the acylation of the ubiquitin C-terminus and subsequent thioesterification, releasing adenosine monophosphate (AMP) in the process [10]. Ubiquitin is transferred to an E2 ubiquitin conjugating enzyme via transthioesterification [11]. Finally the activated ubiquitin is canonically transferred via an E3 ubiquitin ligase to a lysine group on a target protein [9]. The addition of four ubiquitin molecules to the target protein is the canonical signal for transfer of that protein to the 26 kDa proteasome for degradation, but other non-canonical ubiquitination patterns have also been reported [12]. E3 ligases, e.g., muscle specific ring finger protein 1 (MuRF1) and atrogin1, have been the focus of much work after they were found to be elevated in several models of skeletal muscle atrophy [13, 14].

The UPP alone cannot degrade intact myofibrils [15, 16]. Thus, there is a requirement for involvement of one or both of the other protein catabolic pathways, depending on the physiological situation. In terms of degrading sarcomeric proteins, it is believed that the calpain system (further described below) is required to break up sarcomeres into their component parts through the proteolytic activity of the calpains [17]. Much as the UPP and calpain systems are intricately linked to drive the destruction of specific proteins, so too is the autophagy pathway linked to the UPP [18]. Generally, the autophagy system involves the initial generation of an autophagosome surrounding bulk intracellular components or protein complexes. These components targeted for destruction could be intracellular organelles, damaged proteins or other target proteins (usually membrane bound proteins). The autophagosome then fuses with lysosomes leading to the degradation of the autophagosome contents. Several stressors activate autophagy in skeletal muscle, including reactive oxygen species generation and starvation.

The first step in the prototypical autophagy process is the formation of a nascent membrane structure, the phagophore. The origin of the membrane—whether endosomal, trans-Golgi, nuclear membrane or de novo synthesis—is unclear. After the maturation of the autophagosome there is a fusion with lysosomes generating an autolysosome. Finally, activation of lysosomal proteases leads to the degradation of autolysosome contents and the recycling of amino acids. Thus, this system, in combination with the UPP, degrades proteins important for exercise performance and adaptation other than myofibrillar proteins, such as membrane bound proteins, e.g., transporters, ion channels, and receptors [19].

Calpains are non-lysosomal Ca^2+^-dependent cysteine proteases. Candidate targets for calpain activity in muscle include myofibrillar, cytoskeletal, and sarcolemmal proteins. There are three calpains expressed in skeletal muscle; calpain-1, calpain-2 (the ubiquitous calpains), and the muscle-specific calpain-3 [20]. Whereas calpain-1 and 2 require autolysation and heterodimerization with calpain-4, calpain-3 requires autolytic cleavage for activity but not dimerization to calpain-4. Once activated, calpain-1 and calpain-2 are referred to as µ- or m-calpains, respectively, due to their reliance on micro- or millimolar concentrations of Ca^2+^ for activation. The requirement for millimolar Ca^2+^ levels for activation makes it difficult to discern a physiological role for m-calpain in skeletal muscle. Approximately 70% of µ-calpain is thought to be freely available in the cytoplasm of skeletal muscle. Upon a rise in [Ca^2+^], calpain-1 dimerizes with calpain-4 and binds to target proteins. Further sustained increases in [Ca^2+^] are required for activation to µ-calpain and the dissociation between target binding and activation is thought to be a mechanism to prevent inappropriate calpain driven proteolysis [21]. The ubiquitous calpains also are regulated by calpastatin. This regulation requires the heterodimeric form of the calpains and the presence of calcium [22]. Unlike calpain-1, calpain-3 is thought to be mostly bound to myofibrillar proteins, and in particular to titin [23]. The importance of calpain-3 in skeletal muscle homeostasis is underlined by the fact that lack of calpain-3 leads to limb-girdle muscular dystrophy type 2A with sufferers becoming wheelchair-bound from early adulthood onwards [24].

It is clear that the three main protein degradation systems work simultaneously to contribute to the overall response of MPB in response to exercise and nutrition. Whereas mechanistic data are lacking from human studies, data from animal models suggest that the UPP and calpain systems play a much larger role than autophagy [17]. However, the autophagy system seems to be particularly important for degradation of receptor proteins at the membrane [19]. Given their importance in control of anabolic processes, control of receptor protein degradation plays an important role in muscle remodeling. Assessing markers of these pathways offers important mechanistic information leading to greater understanding of the role of MPB in muscle remodeling in response to exercise and nutrition.

## Methods for Measuring Muscle Protein Breakdown (MPB)

Methods to assess the response of MPB to exercise and nutrition interventions in humans can be divided broadly into dynamic and static measurements. Measurements of the dynamic response of MPB are based primarily, albeit not entirely, on stable isotopic tracer methods. Static measurements stem primarily from assessing changes in molecular signals in muscle biopsy samples. All methods have their strengths and limitations. These considerations must be balanced with the level of invasiveness required for each method when choosing how best to assess the response of MPB. It is important to understand the strengths and limitations of methods used to measure MPB for optimal interpretation of the available data. We attempt to delineate these considerations for the methods discussed below.

### Dynamic Measures of MPB

Stable isotopic tracer methods provide a powerful tool to determine metabolic responses to various perturbations, including nutrition and exercise. Arteriovenous (AV) blood sampling in combination with infusion of stable isotopically labeled amino acids has been used to assess MPS and MPB in vivo in humans [25]. This two-pool (arterial and venous amino acid pools) model allows calculation of the uptake and release of an amino acid that is not metabolized in muscle (such as phenylalanine) across the limb [26, 27]. The uptake and release are assumed to be due directly to MPS and MPB, respectively. For release of phenylalanine from the leg to represent MPB, it must be assumed that outward amino acid transport from the muscle into the venous pool is equivalent to MPB and both processes are in steady state [26, 27]. So, MPB may be underestimated by the amount of amino acids that appear in the muscle intracellular pool that are reutilized for MPS and not transported out into the venous blood [28, 29]. Also, both physiological and isotopic steady-states are necessary for this model to offer robust results [26, 27, 30]. However, in many nutrition and exercise studies, physiological steady state is not possible. For example, when a bolus ingestion of amino acids or protein is a necessary component of the study design, transient expansion of the intracellular amino acid pool followed by amino acid efflux into the venous blood pool will result [31]. This transient expansion must be accounted for when calculating MPB. Thus, measurement of MPB in these situations is less reliable. Therefore, this two-pool model for estimation of MPB may be reliable and useful in certain situations, e.g., studies in which physiological and isotopic steady states are possible. However, the limitations must be considered carefully, particularly in studies involving bolus ingestion of a source of amino acids following exercise.

An important limitation of the two-pool AV balance model that must be considered is that it underestimates the true rate of MPB depending on the rate of amino acid transport, as well as the reutilization of intracellular amino acids for MPS. Thus, more recently an AV model was developed to determine the actual rate of appearance of amino acids into the intracellular pool from MPB [28, 29]. This three-pool (arterial, venous, and muscle intracellular) model provides a closer approximation of the true rate of MPB. In addition to the arterial and venous blood samples, the isotopic enrichment of intracellular amino acid tracers is determined from muscle biopsy samples. As with the two-pool model, both physiological and isotopic steady state are assumed with this three-pool model [28, 29]. Thus, the three-pool model is a refinement of the two-pool AV model, but limitations remain.

The AV balance models have been used to provide important information about muscle metabolism following exercise, including MPB. Yet, the limitations inherent with these models require careful consideration when interpreting results. The most commonly used limbs are the leg and forearm. Since samples are taken from venous blood draining an entire limb (the femoral vein drains the entire leg, not just the muscle tissue) calculation of MPB includes contributions from non-muscle tissues (e.g., skin, bone etc.). Biolo et al. [29] determined that muscle accounts for 85–90% of the metabolism of the leg at rest [3, 7]. The contribution of non-muscle tissue is likely more for the forearm [3]. Given that exercise increases the metabolism of the muscle with little impact on other tissues, it is a reasonable assumption that measurements made using these AV models following exercise represent changes in muscle metabolism [3, 7]. Moreover, MPS calculated as the fractional synthetic rate (FSR)—a method that measures metabolism only in muscle tissue—is highly correlated with MPS determined by the three-pool AV model [7] suggesting that muscle metabolism is the primary contributor to the results. Another obvious consideration is the invasive nature of sampling from an artery and vein that drains an entire limb. Whereas any artery may be sampled, an appropriate vein must be sampled, e.g., femoral vein for leg AV balance. Catheterization of an artery and a deep forearm or femoral vein obviously must be performed with great care and under appropriate clinical conditions and ethical considerations. Thus, utilization of these models is limited mostly to clinical facilities making these methods largely unavailable for studies in healthy volunteers—both athletes and other exercisers.

Other stable isotopic tracer models have been developed to assess MPB in vivo in humans when arterial catherization may not be feasible [32]. The principle behind these methods is that the appearance of unlabeled amino acids from MPB will dilute the tracer enrichment in the muscle intracellular pool, but not arterial blood pool [33]. So, the relationship of the enrichment in the muscle intracellular fluid and arterialized blood can be used to calculate fractional breakdown rate (FBR), i.e., MPB [32]. An advantage to this model is that MPS can be simultaneously determined and NBAL calculated. FBR can be determined simultaneously with FSR by infusing two isotopes and sampling muscle tissue and arterial or arterialized venous blood [32, 34, 35]. Whereas arterial catherization is not necessary, two or more muscle biopsies are required. More recently, a pulse-bolus version for determination of FBR was developed in the Wolfe laboratory [36]. This method requires fewer biopsies and does not require an infusion of amino acids. Physiological steady state is a crucial component of these models to determine FBR. Without this steady state, such as occurs with ingestion of a source of amino acids, the relationship between MPB and amino acid transport is variable [37] and the model breaks down leading to unreliable results. Thus, it is not possible to determine MPB following exercise with ingestion of protein or amino acids using these models. Recently, a technique was developed to address this shortcoming [37], but to date it has never been validated in humans. So, to our knowledge, it is possible to measure MPB in response to an infusion or constant ingestion of steady doses of protein or amino acids with AV balance methods. However, assessing the response of MPB to a bolus ingestion of a source of amino acids remains problematic. These limitations have led to the dearth of available data on MPB in humans.

Both AV balance and FBR methods to assess MPB are limited to degradation rates of mixed muscle proteins, i.e., all proteins in the muscle. There is no resolution of breakdown on the individual protein level or even protein subfraction, e.g., myofibrillar versus mitochondrial proteins. Measuring rates of synthesis of protein subfractions has become quite common over the past decade [2, 5, 38, 39]. However, measuring breakdown rates of these protein subfractions is difficult. One approach that attempts to address this limitation is to measure 3-methylhistidine (3MH), which is a post-translationally methylated histidine found in myofibrillar proteins. 3MH is used as a marker of myofibrillar MPB because it cannot be further metabolized nor can it be reutilized for MPS. Many studies have measured urinary 3MH as a marker of whole body myofibrillar protein breakdown. But, of course, 3MH is found in tissues other than skeletal muscle, e.g., cardiac and smooth muscle, so increased urinary 3MH does not represent only skeletal MPB. Moreover, studies measuring 3MH must ensure that participants consume a meat free diet. More recently, AV balance of 3MH has been used to determine MPB, but this method has been used sparingly and only in clinical studies [40]. Interstitial 3MH recently has been measured after exercise and inactivity using microdialysis [41–43]. However, in addition to the above limitations, it must be assumed that increased 3MH in the interstitial fluid appears as a result of increased MPB. This method has been criticized [44] and the sensitivity of the measurement seems to be insufficient to detect changes in MPB following many forms of exercise [43]. Thus, the efficacy of using this method to assess myofibrillar MPB following exercise in humans is uncertain, particularly given the intricacies of muscle microdialysis techniques.

More recently, attempts have been made to investigate breakdown of individual proteins in muscle. One method involves measurement of decay of isotopic enrichment of individual proteins [45, 46]. However, this method has yet to be used in a study involving exercise and nutrition. Moreover, this method measures MPB only over the course of at least several days and as much as 2–3 weeks. Thus, the rates of MPB calculated would not be comparable to acute measurements of MPS. Whereas the time frame of MPB measurement may be applicable to what can be generated with deuterated water methods for assessing MPS [47, 48], the two methods may not be used simultaneously and NBAL cannot be determined. So, the utility of this method for assessing breakdown of individual proteins [45] seems to be fairly limited.

Another method recently has been developed to determine breakdown of individual proteins in muscle using proteomic analysis. Breakdown rate of each protein is calculated from the measurement of the synthesis of each protein using stable isotopic tracers combined with changes in abundance of the protein. This method has been reported in fish [49] and, more recently, in humans following exercise during low carbohydrate, high fat feeding [50]. Since determination of MPB is indirect, there are limitations that must be considered. Breakdown rates of some proteins have been reported to be negative due to a number of factors [50]. Thus, whereas this method offers a way to acquire some important information about the response of MPB to exercise and nutrition, appropriate caution should be applied with interpretation of the results.

In summary, there are a number of methods that have been used to determine the dynamic response of MPB to exercise and nutrition. Most of these methods utilize stable isotopic tracer techniques to assess MPB and the various limitations of the available methods make measurement of MPB much more difficult than MPS. Thus, there is much less information on the response of MPB to exercise and nutrition available. Nevertheless, important mechanistic information may be gleaned from these studies.

### Molecular Pathways of MPB

Information about the response of MPB in humans also may be gleaned from measurements of changes in the response of molecular signaling of MPB pathways. These measures are made from muscle samples taken at a given, individual time point. Thus, these assessments of MPB pathways in response to exercise are mainly generated through examination of ribonucleic acid (RNA) or protein levels or indices of protein signaling/activation responses (phosphorylation, autolysation, etc). The development of the polymerase chain (PCR) methodology and subsequently quantitative real-time PCR (qRT-PCR) led to an explosion of studies using these techniques to assess gene expression [51]. Whilst qRT-PCR takes a gene by gene approach, global scale technologies such as microarrays [52] and more recently RNA-sequencing (RNA-Seq) [53] also are available to quantify the transcriptional response of muscle to exercise. The sensitivity of these molecular biology methods means that great care should be taken with sample preparation to prevent contamination with exogenous RNA that can confound findings. This sensitivity also means that very small amounts of tissue are required. RNA levels can be assessed alongside other parameters (e.g., protein levels, enzyme activity) in the same tissue sample. Human exercise studies most commonly measure changes in messenger RNA (mRNA) expression of E3 ligases, e.g., MuRF1 and atrogin1, to suggest changes in MPB with various interventions [6].

A major weakness of any assessment of RNA levels is that these do not always reflect physiological changes in muscle metabolism or mass [54]. For example, Reitelseder et al. [55] reported no change in MPB rates measured with stable isotopic tracer methods, but MuRF1 expression was increased following exercise. Additionally, calpain-3 mRNA levels were reduced 24 h after eccentric exercise [56], whilst calpain-3 autolysis, and presumably activity level, was increased after eccentric exercise at the same time point [57]. Thus, increased mRNA expression does not always point to increased activity of a pathway. Furthermore, the quantification of RNA levels by qRT-PCR is usually relative and so the absolute level of RNA species examined is usually unknown. Finally, the response of mRNA expression of multiple proteins may be variable [55, 58–60] making interpretation of these results problematic. These variable responses may suggest different functional properties of the proteins. Nonetheless, these limitations must be carefully considered when results using these methods are appraised.

When using qRT-PCR, levels of the target RNA are usually compared to a normalizer RNA, which should not change across conditions. Other normalization methods also are available [61]. The unchanging nature of this normalizer RNA often is not examined explicitly and the choice of reference gene to use as a normalizer is often accepted without critical evaluation [62]. Studies have examined suitable panels of reference genes to use for qRT-PCR in skeletal muscle [63] and tools have been developed to help researchers select suitable normalizer genes for a qRT-PCR experiment [64]. Partly in response to low reproducibility rates, guidelines for adequate reporting of qRT-PCR studies were published [65]. Along with several other excellent recommendations these guidelines include the explicit checking of normalizer RNA expression stability and level. Indeed, it is probably optimal to use more than one normalizer RNA and appropriate normalization methods [66, 67]. Nonetheless for interpretation of studies of MPB or atrophy that use qRT-PCR the reader should be aware that uncritical use of ‘stock’ normalizing RNA species is still widespread. Studies examining the expression of ‘usual’ normalizer RNAs for qRT-PCR are rare but Sunderland et al. [68] reported that the expression of several usual normalizer RNAs can be influenced by both subject age and time after exercise.

Microarrays [52] and RNA-Seq [53] both give a global overview of transcription and this information can be used to examine enriched pathways and processes or to identify potential markers for high or low responders. The advantage of these technologies is the broad coverage of transcriptional activity for the RNA species of interest. Microarrays and RNA-Seq also can be adapted to give information on the epigenetic state of DNA (i.e., methylation, acetylation, etc.). Microarray and RNA-Seq are both very sensitive to contamination and as with qRT-PCR, care must be taken in sample preparation. However, whilst functional events at the protein level cannot be directly inferred, the global nature of the profiling does mean that the biological context of the tissue can be inferred. The global methods can return large numbers (possibly thousands) of genes or other entities that vary with the condition of interest and making sense of these lists is challenging. The most widely adopted approach is one of enrichment or category analysis [69]. Enrichment analysis takes a list of identified genes and uses statistical testing to ask if any pre-curated biological process, function or pathway is enriched in that list and, if so, in which direction the genes change with the condition. The prototypical example of this technique is gene set enrichment analysis (GSEA) [70]. Various curated repositories provide information on whether genes belong to biological processes or pathways [71–73]. One caveat with enrichment analysis is that the information in these resources is constantly changing as new findings come to light.

## Response of MPB to Exercise and Nutrition

### Exercise

Exercise is a powerful mediator of MPB. Generally, it is thought that resistance exercise increases MPB [6]. In the first study to assess MPB using dynamic, stable isotopic tracer methods we demonstrated that mixed protein MPB was increased following resistance exercise in untrained volunteers [7]. The increase in MPB was less than the increase in MPS, so NBAL was increased. However, NBAL did not reach net positive balance during these measurements in the fasted state [1]. These MPB results generated using AV balance methodology were replicated subsequently using another stable isotopic method. The FBR of mixed muscle proteins was increased following resistance exercise in untrained individuals, but less than FSR leading to improved, but still negative NBAL [34]. Interestingly, FBR was increased for 24 h following exercise whereas FSR remained elevated for 48 h. Recently, FBR also was reported to be unchanged by resistance exercise 48 h prior during an energy deficit [74]. Thus, it seems clear that, at least with a sufficient stimulus, resistance exercise stimulates increased mixed MPB in untrained volunteers.

Broad support for the notion that MPB is increased following resistance exercise comes from studies measuring molecular markers. Studies consistently report that muscle specific ubiquitin ligase MuRF1 mRNA expression was increased in the first few hours after resistance exercise in untrained individuals [55, 60, 75–79]. However, mRNA expression of atrogin1, another E3-ligase, reportedly decreased [60, 75, 80] or remained unchanged [75, 81] following resistance exercise. Recently, Hector et al. [74] reported that a number of molecular markers of MPB were unchanged 48 h following resistance exercise during energy deficit conditions. These divergent responses suggest the roles of these ligases may vary. Alternately, the response of the two ligases may be dependent on fiber type [12, 77]. It is important to note that these measures come from only a single time point, so they represent a ‘snapshot’ of the response. Moreover, increases in mRNA do not always lead to increased protein levels, not to mention physiological activity. Changes in mRNA expression are often not associated with dynamic measures of MPB [55]. Thus, given that the preponderance of the available data shows that expression of at least some components of the ubiquitin-proteasome pathway increase, overall these results are consistent with the dynamic measurements indicating that MPB increases in response to resistance exercise.

Training status seems to impact the response of MPB to resistance exercise. Using a cross-sectional comparison, we demonstrated that mixed muscle FBR was increased following resistance exercise in untrained individuals [35]. However, the same exercise bout (i.e., same relative exercise intensity) resulted in little, if any, increase in FBR in resistance-trained individuals. Moreover, there was no difference in resting FBR between trained and untrained individuals [35]. Subsequently, FBR was measured using a longitudinal study design before and after 8 weeks of training [82]. Resting FBR was greater following than before training. Moreover, resistance exercise increased FBR prior to training, but not after training. It should be noted that FBR was measured following exercise at the same absolute exercise intensity and during constant feeding in this study [82]. So, it is difficult to compare these results directly to the previous results [35]. On the other hand, taken together these results from different studies under varying physiological conditions support the notion that training reduces the response of MPB to resistance exercise. Stefanetti et al. [76] showed reduced MuRF1 expression with resistance exercise following 10 weeks of resistance training. This response contradicts that demonstrated in untrained individuals [55, 60, 75–79]. It is generally assumed that the response of global MPB to resistance exercise reflects the degradation of myofibrillar proteins.

There have been attempts to refine the measurement of MPB to the breakdown of the myofibrillar protein fraction. Since 3MH is found only in myofibrillar proteins, measurement of 3MH in the muscle interstitial fluid using microdialysis techniques has been used to assess myofibrillar protein degradation. These studies report no change in interstitial 3MH following resistance exercise [41, 43]. Similarly, intense endurance exercise did not result in increased interstitial 3MH [42]. These results [42, 43, 83] may be interpreted to suggest that myofibrillar breakdown is not a major contributor to the increase in mixed MPB due to intense exercise [7, 34, 35]. However, one study demonstrated that interstitial 3MH increased in response to electrical stimulation, but not intense eccentric contractions [43], similar to that previously shown to increase mixed MPB [7, 34, 35]. This discrepancy suggests that measurement of interstitial 3MH likely is not sensitive enough to detect changes in myofibrillar protein breakdown following resistance exercise [43]. Moreover, the use and validity of this methodology has been criticized and the results questioned [44]. Thus, whereas it is intuitively satisfying to believe that degradation of myofibrillar proteins provides a major proportion of overall MPB following exercise, the precise contribution of this protein fraction to overall MPB after exercise remains to be fully elucidated.

There is even less known about the dynamic response of MPB to endurance exercise compared to resistance exercise. Early reports of increased 3MH excretion suggest that myofibrillar MPB is increased by endurance exercise [84]. More recently, AV balance measurements showed that MPB was increased at 10 min, but not 60 or 180 min, following 45 min of walking on a treadmill [85]. A recent study showed no change in FBR following 45 min of running at 65% *V*O_2peak_ [86], but the determination of MPB may have been confounded by the fact that it was measured in the vastus lateralis muscle in trained volunteers. Molecular indicators of MPB have been reported to increase in response to endurance exercise [59, 76, 80, 87–89]. Thus, the consensus seems to be that resistance exercise stimulates an increase in MPB, but it is not clear what the response is following endurance exercise. Clearly, more studies need to focus on the response of MPB, particularly the dynamic physiological response, to exercise of various types.

### Combination of Nutrition and Exercise

The role of MPB in the response of NBAL following resistance exercise and nutrition is somewhat controversial [2, 90]. Whereas the response of MPS to protein nutrition and exercise has been studied extensively [2–5], there are methodological difficulties that make measuring the response of MPB to exercise and nutrition problematic. The available information comes primarily from AV balance studies. Biolo et al. [8] infused amino acids systemically following a resistance exercise bout and used the three-pool AV balance model to assess muscle protein metabolism. MPS was increased during hyperaminoacidemia following exercise, but there was no increase in MPB compared to resting, fasted levels. Similarly, the combined ingestion of essential amino acids and carbohydrate prevented exercise-induced MPB [91]. Unfortunately, the available, albeit limited, molecular data do not shed much light on these responses. Branched-chain amino acids (BCAA) [9, 92], intact protein [55, 81] and essential amino acids [91] seem to have no impact on MuRF1 expression. However, there is one report of reduced atrogin1 expression with post exercise BCAA ingestion [75]. It may be that the response of UPP expression is influenced by the dose of protein ingestion. Areta et al. [58] reported increased MuRF1 expression following exercise with ingestion of 10 and 20 g of whey protein. However, ingestion of 40 g prevented the increase in mRNA levels. Unfortunately, it is unclear how these changes in mRNA levels relate to changes in MPB rates [54]. Nevertheless, it seems that hyperaminoacidemia, possibly mediated primarily by BCAA, inhibits the increase in MPB following exercise.

As with hyperaminoacidemia, hyperinsulinemia inhibits the increase in MPB following resistance exercise [91, 93]. However, no increase in MPS has been reported in response to hyperinsulinemia following exercise [91, 94, 95]. Thus, improved NBAL with carbohydrate ingestion following resistance exercise stems almost entirely from inhibited MPB. However, it should be noted that no determination of the proteins involved has ever been made. It is clear that increased synthesis of myofibrillar proteins results from resistance exercise, alone and with ingestion of amino acids [39]. Nevertheless, there is no evidence that myofibrillar protein breakdown is increased with resistance exercise [41, 43] or that hyperinsulinemia impacts any particular protein or protein fraction [96]. Thus, the changes in synthesis and breakdown due to exercise in combination with hyperinsulinemia and hyperaminoacidemia may impact completely different proteins. Consequently, the mathematical calculation of NBAL may not offer much important information. At this point, there is no way to determine the physiological relevance of this calculation in terms of the response to insulin and amino acids.

The response of MPB to exercise also has been investigated during periods of reduced energy intake resulting in an energy deficit. A 20% energy deficit in healthy, physically active young males and females resulted in an ~ 60% decrease in MPB assessed by FBR [86]. Most molecular markers, e.g., mean chymotrypsin-like activity, expression of atrogin-1, of MPB were unaltered by energy deficit, but caspase-3 activity was ~ 11% greater than during energy balance. Alternatively, Hector et al. [74] reported that 40% energy deficit did not alter MPB (FBR) in young, overweight males. Further, no change in molecular markers of MPB was reported. The reason for the differences in these results is not certain, but may be related to the participant characteristics [74]. Nevertheless, there was no response of MPB to exercise, 45 min of running [86], or 48 h after resistance exercise [74], during energy deficit in either study. As with other nutrition and exercise situations, the paucity of studies on this topic limit a firm conclusion about the role of MPB during energy deficit at this juncture.

This response of MPB to nutrition and exercise may be explained by the physiological relationship of MPS and MPB. Resistance exercise increases MPS [7, 34, 35], likely mediated by the mammalian target of rapamycin 1 (mTORC1) signaling pathway [38, 97]. Thus, there is increased demand for intracellular free amino acids to supply substrate for the increased rate of MPS. Without an exogenous source of amino acids, amino acid availability for MPS is limited and MPB is increased to supply the amino acids [3]. The fact that MPS and MPB are highly correlated when measured following exercise in the post-absorptive state [3, 7, 34, 35] supports this notion. However, when amino acid availability is increased by a source of exogenous amino acids, there is no need for MPB to increase to supply amino acids for increased MPS [3, 7, 34, 35].

## Future Directions

**Fig. 1:**
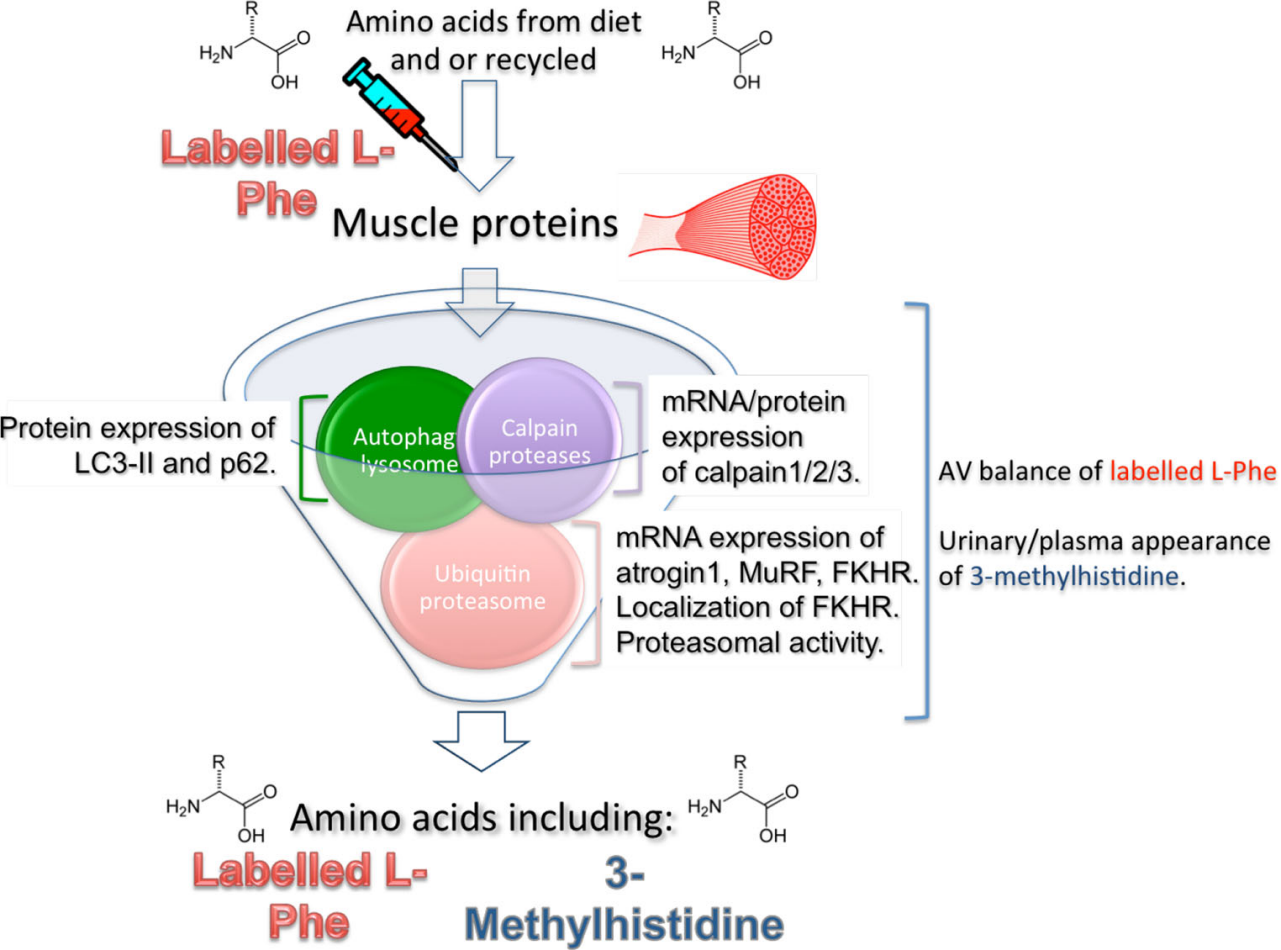
Methods of assessing skeletal muscle protein breakdown (MPB). Skeletal muscle proteins are broken down by a combination of the three main protein breakdown systems. These breakdown systems do not work in isolation but rather work together to remodel skeletal muscle. (1) The calpain proteases disassemble myofibrils into smaller component parts, (2) the ubiquitin-proteasome system degrades these component into individual amino acids, and can label proteins (membrane receptors, channels and transporters) for destruction by the third system, (3) the autophagy-lysosome system, which predominantly breaks down membrane based proteins. Dynamic MPB measures use labelled amino-acid tracers (such as phenylalanine stable-isotopes) and provide a dynamic view of whole MPB. 3-Methylhistidine is a unique metabolite of myofibrillar protein breakdown and its appearance in blood and urine can be assumed to have come from the processes of myofibrillar protein breakdown. Skeletal muscle is the body’s largest depot of myofibrillar protein so changes in plasma/urinary/interstitial 3-methylhistidine are believed reflective of skeletal MPB. Other static markers of protein breakdown include the assessment of the messenger RNA (mRNA)/protein expression/activity/localization of components of the breakdown machinery. Markers are available to estimate changes in the activity of each of the three breakdown systems. *L-Phe* l-phenylalanine, *AV* arterio-venous, *MuRF* muscle ring finger protein, *FKHR* forkhead transcription factor

It seems clear that our understanding of the response of MPB to exercise and nutrition is incomplete. There are promising new techniques to assess the dynamic response of MPB [37, 45] that need to be validated in various physiological situations, including post exercise with nutrient ingestion. Simultaneous measurements of MPB rates using dynamic, stable isotopic tracer methods and static markers of MPB pathways may provide important mechanistic data to enhance our understanding. However, it must be stressed that the individual components of the machinery responsible for driving MPB do not work in isolation. Rather, each component is linked and changes in one component in isolation may or may not be responsible for driving a change in overall MPB as assessed by dynamic measures (Fig. 1). That said, the additional information that may be gleaned from studies combining these techniques in an integrated manner may add a great deal to our understanding of the contribution of the various components of the MPB machinery to changes in muscle mass, as well as muscle remodeling and adaptations to training.

## Conclusions

MPB is a critical aspect of the response of muscle metabolism to an exercise bout, as well as adaptations to training. Changes in the amount of any particular protein ultimately result from the balance between the rate of synthesis and breakdown of that protein over any given time. We know that nutrition can suppress MPB following exercise [8, 91, 93]. As such, recommendations for nutritional interventions that inhibit MPB often are made. It is assumed that suppression of MPB following resistance exercise will contribute to increased NBAL and thus increased muscle mass [3, 4]. That assumption would be true if all of the inhibition was of intact, undamaged myofibrillar proteins. However, at least some of the measured global MPB resulting from exercise likely represents degradation of damaged proteins and/or proteins with rapid turnover. Degradation of these proteins likely is an important part of the adaptive process for remodeling and reconditioning muscle proteins. Thus, nutrition interventions resulting in inhibition of degradation of unnecessary or damaged proteins may actually impair adaptation to exercise training.

We simply do not know enough about the response of various individual proteins to exercise of various types. Moreover, we know next to nothing about how various nutrition interventions impact the degradation of particular proteins. Therefore, it may be a mistake to attempt to limit MPB with nutritional interventions following exercise. Finally, the changes in MPS are much greater than in MPB following exercise [5, 8, 34]. Taken together, at least until we accumulate more information on the role of degradation of various proteins in muscle remodeling, nutrition recommendations to enhance training adaptations most likely should focus primarily on the response of MPS.

Nevertheless, information on the response of MPB to exercise and nutrition provides critical information toward our understanding of muscle metabolism and exercise, as well as the influence of exercise variables and nutrition on training adaptations. This information may be useful, not only to athletes and other exercisers, but also overall metabolic health and mortality. Unfortunately, at least in humans in vivo, the technical difficulties of measuring MPB limit our current understanding of these processes. New methods for assessing MPB in various situations, including for example bolus ingestion of proteins following exercise, will be critical for evaluating the importance of changes in MPB, as well as the precise contributions of these mechanisms to muscle metabolism.
